# Supplementary material for: End Sequence Analysis Toolkit (ESAT) expands the extractable information from single-cell RNA-seq data
Source: Genome Res. 2016 Oct;26(10):1397–410. doi: 10.1101/gr.207902.116 (PMC5052061; doi:10.1101/gr.207902.116)
Supplement: Supplemental Material [file supp_gr.207902.116_Supp_Materials.tar.gz › ESAT-esat_v0.1/doc/html/classbroad_1_1core_1_1primer3_1_1is_p_c_r_like.html]

ESAT: broad.core.primer3.isPCRLike Class Reference


ESAT


- Main Page
- Related Pages
- Packages
- Classes
- Files

- Class List
- Class Index
- Class Hierarchy
- Class Members

All Classes Namespaces Files Functions Variables Enumerator Pages

- broad
- core
- primer3
- isPCRLike

Public Member Functions |
Static Public Member Functions |
List of all members

broad.core.primer3.isPCRLike Class Reference

Collaboration diagram for broad.core.primer3.isPCRLike:

[legend]

|  |  |
| --- | --- |
| Public Member Functions | |
|  | isPCRLike (Pair< String > primers, List< Sequence > sequences) |
|  | |
| Collection< Sequence > | getAllPossibleAmplicons () throws SearchException |
|  | |

|  |  |
| --- | --- |
| Static Public Member Functions | |
| static void | main (String[] args) throws IOException, SearchException |
|  | |

## Constructor & Destructor Documentation

|  |  |  |  |
| --- | --- | --- | --- |
| broad.core.primer3.isPCRLike.isPCRLike | ( | Pair< String > | *primers*, |
|  |  | List< Sequence > | *sequences* |
|  | ) |  |  |

Here is the caller graph for this function:

## Member Function Documentation

|  |  |  |  |  |
| --- | --- | --- | --- | --- |
| Collection<Sequence> broad.core.primer3.isPCRLike.getAllPossibleAmplicons | ( |  | ) | throws SearchException |

Get all possible amplicons as subsequences of the original sequences Name of subsequence is equal to the name of the parent sequence

Returns
:   Collection of possible amplicons

Exceptions
:   |  |  |
    | --- | --- |
    | SearchException |  |

|  |  |  |  |  |  |
| --- | --- | --- | --- | --- | --- |
| static void broad.core.primer3.isPCRLike.main | ( | String[] | *args* | ) | throws IOException, SearchException |

static

Here is the call graph for this function:

---

The documentation for this class was generated from the following file:

- java/broad/core/primer3/isPCRLike.java


---

Generated on Fri Aug 15 2014 15:10:55 for ESAT by  

 1.8.7
